# Electrochemical Kinetics and Detection of Paracetamol by Stevensite-Modified Carbon Paste Electrode in Biological Fluids and Pharmaceutical Formulations

**DOI:** 10.3390/ijms241411269

**Published:** 2023-07-10

**Authors:** Moaad Gharous, Loubna Bounab, Fernando J. Pereira, Mohamed Choukairi, Roberto López, A. Javier Aller

**Affiliations:** 1 Laboratory of Materials and Interfacial Systems, Faculty of Science, Abdelmalek Essaadi University, BP 2121, Tetouan 93002, Morocco; moad.gharous@etu.uae.ac.ma; 2 Research Group of Advanced Materials, Structures and Civil Engineering, National School of Applied Sciences of Tetouan, Abdelmalek Essaadi University, BP 2121, Tetouan 93002, Morocco; lbounab@uae.ac.ma; 3 Department of Applied Chemistry and Physics, Faculty of Biological and Environmental Sciences, Campus de Vegazana, s/n, University of León, E-24071 León, Spain; rlopg@unileon.es (R.L.); aj.aller@unileon.es (A.J.A.)

**Keywords:** paracetamol, chemically modified electrode, voltammetric techniques, clays, carbon paste electrodes

## Abstract

Paracetamol (PCT), or acetaminophen, is an important drug used worldwide for various clinical purposes. However, the excessive or indiscriminate use of PCT can provoke liver and kidney dysfunction; hence, it is essential to determine the amount of this target in biological samples. In this work, we develop a quick, simple, and sensitive voltammetric method using chemically modified electrodes to determine PCT in complex matrices, including human serum and commercial solid formulations. We modify the carbon paste electrode with stevensite monoclinic clay mineral (Stv-CPE), using cyclic voltammetry, differential pulse voltammetry, and electrochemical impedance spectroscopy to characterise and detect PCT. The kinetics study provides a better electrochemical characterisation of the electrode behaviour, finding the detection and quantitation limits of 0.2 μM and 0.5 μM under favourable conditions. Further, the best linear working concentration range is 0.6–100 μM for PCT, applying the proposed method to the quantitative determination of PCT content in reference tablet formulations and biological samples for validation.

## 1. Introduction

Paracetamol (PCT), known as acetaminophen (*N*-acetyl-*p*-aminophenol), is one of the public’s most widely recognised chemical antipyretic and non-steroidal anti-inflammatory drugs [1]. PCT is included in the composition of several formulations for treating fever of viral and bacterial origin, cough, cold, muscle pain, chronic pain, migraine, headache, backache, and toothache [1]. The prescription of PCT-based drugs is frequent worldwide because it has no harmful side effects under recommended consumption. However, overdose and chronic use of PCT produce an accumulation of toxic metabolites that can lead to kidney and liver failure [2,3]. Furthermore, PCT arrives in the environment through sewage plant effluents, sewage sludge, hospital wastewater, surface water, and drinking water [4]. All of these considerations call for new performance analytical means to determine PCT and other drug molecules.

The analytical methodologies used to determine PCT involve diverse techniques, particularly liquid chromatography, gas chromatography, spectrophotometry, spectrofluorometry, chemiluminescence, and mass spectrometry [5,6,7,8]. However, many require sophisticated and expensive instruments, laborious methodologies, time, and experienced personnel [9]. Nonetheless, electrochemical techniques avoid many drawbacks, for which carbon paste electrode (CPE)-based chemically modified electrodes can meet these advantageous needs [10]. It is usual to consider chemically modified electrodes the most attractive and promising technology owing to their ease of use, fast response, low cost, wide linear range, high sensitivity, and low detection limits [11].

Among chemically modified electrodes, modified CPE, mainly including amperometric detection, are inexpensive, easy to prepare, renewable, and reproducible [12]. The principle focuses on a redox reaction or a change in electrical conductivity at the liquid/solid interface. These electrodes have a sensitive layer, acting as the essential part of a transducer system, that transforms the chemical reaction into an electrical signal, detectable and interpretable using voltammetric techniques. Recently, CPEs have integrated diverse particulate materials and primary synthetic nanoparticles with varied compositions [13,14,15,16,17,18], where different metal oxides [12,19,20,21,22,23,24,25,26,27,28], nanocomposites [29], zeolites [30,31], dyes [32] and clay minerals [33] have attracted significant attention to preparing CPE-based chemically modified electrodes [34,35,36,37,38]. On the other hand, clays are minerals dominantly made from a colloid system containing soils, sediments, rocks, and water [39,40]. These materials attracted scientific attention as electrode modifiers because of their exceptional properties, good thermal, mechanical and chemical stabilities, high porosity, and excellent adsorption and exchange capacities, which impart changes in the electrical conductivity and catalytic activity towards electrochemical processes [40].

In addition, the mechanistic oxidation process of PCT, widely accepted as two electron-two protons, assumed *N*-acetyl-*p*-benzoquinone-imine as a first oxidation product, firstly described in 1981 [41] and later also evaluated by other authors [42,43,44,45], could change with the electrode composition and experimental conditions, which is still not definitively resolved and requires further investigation. Generally, PCT oxidation is an irreversible process requiring high overpotentials, which decreases the analytical sensitivity [45]. For this reason, working electrodes are chemically modified with electrocatalysts to solve this problem, reduce overpotential, and improve the electron transfer rate [43]. Under these circumstances, PCT’s electrochemical oxidation usually becomes a quasi-reversible process. In any case, the electrochemical oxidation process of PCT might change with the solution pH, which changes the potential peak and the peak current intensity. The modified CPE have been widely used for the detection of PCT in several works; these sensors have shown electroanalytical performance such as a wide linear range, high sensitivity and stability of recorded signals, and very clear selectivity.

This work aims to elaborate and evaluate, for the first time, the electrochemical behaviour of PCT using Stevensite (Stv) monoclinic (Rhassoul or Ghassoul) clay mineral as a modifier of CPE. A second proposal is to develop a simple, rapid, robust, and sensitive voltammetric approach for determining PCT in pharmaceutical formulations and biological samples in the presence of dopamine (DA) and tyrosine (Tyr) as interferences. The advantages of this novel electrode relate to its capability to work under mild chemical conditions and its easy accessibility to any general laboratory.

## 2. Results and Discussion

### 2.1. Spectrochemical Characterisation of the Rhassoul Clay Mineral

Stv clay is a mineral from the Moroccan Atlas Mountains, containing a high percentage of silica and fewer amounts of magnesium, aluminium, iron, and calcium (Table S1). For the characterisation of this mineral, we used FT-IR spectroscopy (Figure S1) and the complementary techniques SEM/EDS and TEM (Figure S2). Figure S1 shows an FT-IR spectrum of the Stv clay, confirming the presence of silica as the most abundant component. Thus, an intense band centred at 961 cm^−1^ was related to the Si-O stretching vibration, a shoulder at 878 cm^−1^ was ascribed to Si-OH, and a weak peak at 652 cm^−1^ corresponded to the Si-O-Si bending vibration [46,47,48,49]. A broad shoulder centred at 1090 cm^−1^ was ascribed to Al-OH [50]. Additionally, hydroxyl groups (3371 and 3596 cm^−1^) [51,52] facilitated the adsorption properties. It should also be noted that the two weak signals indicated the presence of magnesium at 671 and 726 cm^−1^, both related to the bending vibration of the Mg-OH bond [46].

The Stv clay material prepared for the CPE showed a very small granulometry (Figure S2a). Thus, although SEM micrographs showed some agglomeration of the particles (Figure S2a), the average particle sizes from TEM micrographs were about 50 nm (Figure S2b). The SEM/EDS results also revealed the silicate structure of the Stv and the high amount of magnesium it contained (Figure S2c, Table S1), as it also revealed the FT-IR spectrum. However, EDS spectra showed minor levels of other elements (Figure S2c, Table S1), which improved the prepared electrode’s electrical conductivity, enhancing the charge transfer on the electrode surface.

### 2.2. Effect of the Electrode Composition and Operational Conditions

**Figure 1:**
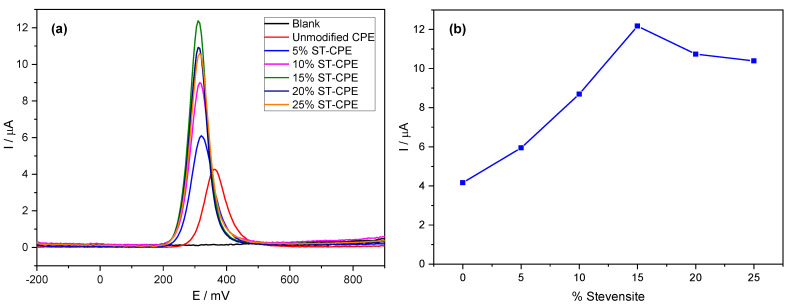
DPV of 200 µM PCT in PBS (0.1 M, pH 6.7) at unmodified CPE and the modified CPE containing 5%, 10%, 15%, 20%, and 25% Stevensite as a function of potential (**a**) and % Stevensite (**b**).

Using the buffer solution (pH 6.7), we found an excellent mass transport rate compared to other buffers. Therefore, we used PBS 0.1 M in all experiments. We investigated the electrochemical behaviour of PCT in a phosphate buffer solution (PBS, pH 6.7) by the analytical techniques CV and DPV (Figure 1). Figure 1a shows the effect of the amount of Stv on the response of the modified CPE toward PCT. The redox peak currents obtained by DPV were much more significant than the ones corresponding to the unmodified CPE. The results from Figure 1a represent evidence of the improved electrocatalytic activity of Stv clay mineral in the CPE towards PCT. The background current of unmodified CPE was smaller than that provided by the modified CPE (with different amounts of clay mineral) for the same amount of PCT, probably due to the large specific area, good conductivity of clay minerals, and a sizeable capacitive current [14]. Further, the peak current grew with the Stv clay content to 15% (Figure 1b), levelling for higher clay mineral contents.

For comparison purposes, we also studied the charge transfer capacity of unmodified CPE and (0–25%) Stv-CPE in a 0.5 M KCl solution containing 0.01 M ${\left[Fe{\left(CN\right)}_{6}\right]}^{3-/4-}$, used as a reference. The impedance diagrams obtained for the reversible system ferri-/ferro-cyanide are composed of two parts (Figure S3): a semicircle in the high frequencies, characteristic of charge transfer, followed by a straight line with a slope close to unity in the low frequencies, which corresponds to the transport of matter (diffusion). The values of load transfer resistance (*R_ct_*) and capacitance (*C_f_*) by fitting the experimental impedance data using EC-Lab software and Randles equivalent circuits are in Table S2. Table S2 shows that the smaller value of *R_ct_* was obtained with 15% Stevensite, indicating a weak electron transfer reaction from ${\left[Fe{\left(CN\right)}_{6}\right]}^{3-/4-}$ to the unmodified CPE.

Notwithstanding, the accumulation time at modified CPE in a 100 µM solution of PCT also showed a remarkable effect on the peak current, increasing significantly with time and reaching a maximum after an accumulation time of 4 min (Figure S4), indicating as well that the prepared electrode effectively accumulates PCT. The peaks became progressively broader at longer times, with a tendency to form a plateau. We used a waiting time of 4 min in all subsequent experiments. The peak shape in Figure 1 shows good symmetry. However, more electrochemical parameters need evaluation to understand the electrochemical processes at the electrode better.

### 2.3. Effect of Scan Rate

**Figure 2:**
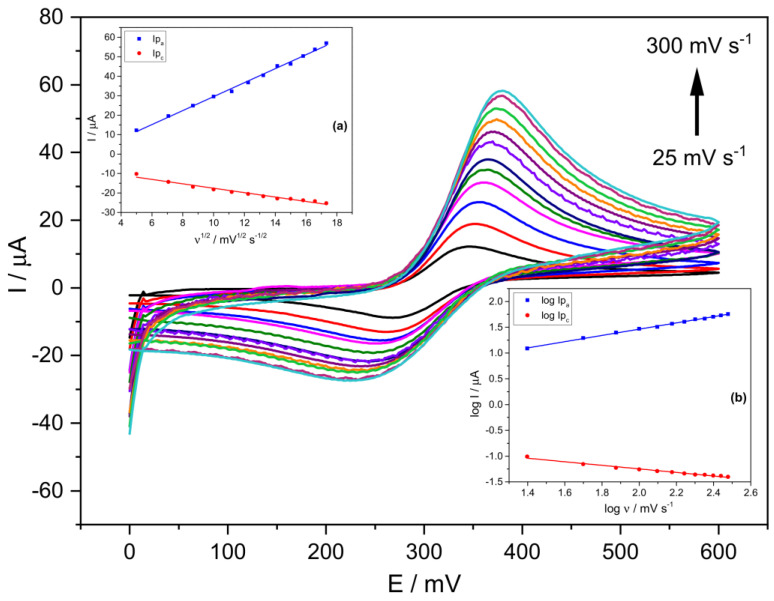
CV voltammograms of a 300 μM PCT in PBS (0.1 M, pH 6.7) and the following scan rates: 25, 50, 75, 100, 125, 150, 175, 200, 225, 250, 275 and 300 (mV s^−1^). (The insets show the linear fittings of (**a**) *I_p_* vs. *ν*^1/2^, (**b**) log *I_p_* vs. log *ν*). The results were obtained from CV voltammograms, employing Stv-CPE of 300 µM PCT solutions in PBS (0.1 M, pH 6.7).

Figure 2 shows cyclic voltammograms using the prepared Stv-CPE electrode with 15% Stv (from now on, Stv-CPE) for various scan rate (*ν*) values between 25 and 300 mV s^−1^ and a 300 μM PCT concentration. The effect of the scan rate on the electrochemical behaviour of PCT solutions with Stv-CPE in PBS at pH 6.7 by CV showed a pair of well-defined redox waves (Figure 2). In the forward scan (positive scan), PCT electrochemically oxidises (anodic process), firstly transforming in *N*-acetyl-*p*-quinoneimine and continuing probably to give *p*-benzoquinone. In contrast, in the reverse (negative) scan, the oxidised-PCT form changes to a reduced form (cathodic process).

It is usual to consider the midpoint potential *E*^0^ or *E_i_* of the anodic and cathodic waves as the standard (formal) potential, which in our case, stays nearly constant with the scan rate range covered (Table 1), with values very similar to those obtained for the half-wave potential *E*_(1/2)_ (Table 1). The standard potential *E*_(*i*)_ represents the half-wave potential *E*_(1/2)_ under standard conditions, being both practically constant since they represent a unique potential for each electrochemical species [53]. Nonetheless, the potential at the anodic and cathodic waves’ inflexion points *E*_(*i*)_ would be identical to *E*_(1/2)_ for reversible processes, which occurs at the lowest scan rates in this work (Table 1). However, slight differences exist between the standard potential *E*_(*i*)_ and the half-wave potential *E*_(1/2)_ for high scan rates, indicating changes in the diffusion layer thickness, which affect the diffusion coefficients of the oxidised and reduced species differently. The following linear relationship {*E*_(1/2)_ = (0.41 ± 0.01) *E_i_* + (−129.0 ± 4.0) (*R*^2^ = 0.99615)} exists between both potentials, showing differences with the increase in the scan rate, and consequently indicating an inevitable irreversibility of the redox process.

The half-wave potentials *E*_(1/2)_ become the best accurate representation of the standard electrochemical potential *E*_(0)_ (or the formal potential, *E*^0^) for reversible electron transfer processes and the same diffusion coefficients for the oxidised and reduced forms. Sometimes, however, the edge potentials (*E*_(*e*)_) of the anodic or cathodic waves estimate quite well *E*_(0)_ for irreversible processes, although they underestimate and overestimate *E*_(0)_ of oxidation and reduction processes [54] (Table 1).

As a whole, the oxidation peak potential of PCT agrees with those provided by diverse previous works for similar CPE-based electrodes, existing as a correlation between the anodic peak and the material type used to modify the CPE composition (Table 2). Thus, the anodic peak of this work correlates well with those found with TiO_2_ and ZrO_2_ materials but differs from those achieved using Fe_2_O_3_, Cu(II)-doped zeolite and graphene, which suggests differences in the redox mechanism on the electrode surface, supported by the differences existing in the peak-potential gap. Thus, the theoretical difference between the anodic and cathodic peaks’ potential (Δ*E_p_*) is 59 mV for a Nernstian behaviour, an ideal one-electron reversible redox reaction and an ideal surface electrode. However, our results show values between 78 mV and 160 mV, showing high response efficiency, possibly due to superior contact with the electrode surface. Improvements are possible in optimising the Stv layer attachment to the transducer surface to improve electrode signal transfer efficiency. However, the other materials used in other works to modify the CPE composition provided essential differences.

Figure 2 shows unsymmetrical peaks with essential differences between the anodic and cathodic processes, dealing with some irreversibility in the electron transfer due to differences in the electrochemical mechanisms involved in the electrode process. We can highlight three main characteristics disclosed in Figure 2. First, the peak potentials change with the scan rate, increasing the anodic peak potential and decreasing the cathodic peak potential. In other words, in the scan rates ranging from 25 to 300 mV s^−1^, the peak-to-peak separation potentials (Δ*E_p_*) of the CV signals increased from ~78 mV at low scan rates to ~162 mV at the highest scan rates (Figure 2 and Figure S5). Second, the peak intensity also varies with the scan rate (Figure S6), indicating changes in the reversibility of the electrochemical system. Third, changes in the electrochemical behaviour of PCT on the Stv-CPE with the scan rate (Figure 2) indicate a quasi-reversible/irreversible process, supported by the intensity (*I_pa_*/*I_pc_*) ratios, which differ from unity and growth with the scan rate, indicating some instability of the oxidation product. Thus, faster scan rates lead to a decrease in the diffusion layer thickness, generating higher currents. Consequently, the electronic exchange does not occur in solution but between a soluble–insoluble system. In addition, no chemical reactions are coupled to the charge transfer step, nor are adsorption phenomena, and the system is not reversible because its behaviour depends on experimental conditions.

On the other hand, Figure S7 shows the plot of the peak potentials vs. ln(*ν*), including three straight lines with slopes different to 60 mV decade^−1^, probably due to a follow-up chemical step, suggesting the existence of some electrochemical irreversibility again. For scan rates below 100 V s^−1^, its behaviour is as expected for a diffusion-controlled process. At the same time, for higher values, the peak potentials move towards more cathodic or anodic values, indicating that the load transfer rate is significantly lower under these conditions than that of mass transfer, changing the reversibility of the system from reversible to quasi- or even irreversible. The peak potential of the irreversible waves changes with the scan rate due to kinetics limitations in the electron transfer at such short times because the electron transfer rate constant is small, needing more extreme potentials to induce electron transfer. At high scan rates, the maximum concentration gradient occurs at potentials further from *E*^0^. Nonetheless, we further included various electrochemical parameters to understand better the PCT electrochemical behaviour in the prepared Stv-CPE.

Ideally, a linear relationship exists between the current and the scan rate, with the plot data’s slope corresponding to the electrode’s capacitance. However, according to the determination coefficient, the theoretical linear relationship between the anodic (or cathodic) current and the scan rate does not fit well. However, a best-fitted linear relationship exists between the oxidation current and the square root of the scan rate (Figure 2, inset (a)), supporting a diffusion mechanism limiting the oxidation process of the analyte, Equation (1), but not for the cathodic process. The poorer fit of the cathodic process, Equation (2), compared with the anodic process, Equation (1), suggests a different and smaller diffusion determination coefficient for the oxidised PCT form. The proper fit between the oxidation peak current and the scan rate’s square root can occur for reversible and irreversible electrode processes (Figure 2, inset (a)), although with different slopes.

*I_pa_* (µA) = (3.59 ± 0.07) *ν*^1/2^ (mV^1/2^ s^−1/2^) + (−6.39 ± 0.90) (*R*^2^ = 0.99616) (1)

*I_pc_* (µA) = (−1.13 ± 0.06) *ν*^1/2^ (mV^1/2^ s^−1/2^) + (−6.20 ± 0.80) (*R*^2^ = 0.97304) (2)

Figure SI-6 shows the plot of $I_{p}/\sqrt{v}$ versus $\sqrt{v}$, where we can note a change in the diffusional regime for values of the scan rate’s square root around ~0.23. At low *ν*, $I_{p}/\sqrt{v}$ occurs an ideal adsorbed species voltammetry and small Δ*E_p_*. At high *ν*, diffusional voltammetry is present, where $I_{p}\;\propto \;\sqrt{\nu }$ (Figure 2, inset (a)). Further, the plot of ln(*I_p_*) versus the peak potential *E_p_* (Figure S8) also shows apparent differences between the anodic and cathodic processes with changes in the *α* value, particularly for the cathodic process. The straight linear relationship between the peak intensity and the potential only occurs around the equilibrium situation (Figure S9).

On the other hand, the logarithm of the anodic peak current also shows a clear linear proportionality to the logarithm of the scan rate (Figure 2, inset (b)), although not so clear for the reduction process, probably because the solution contains diverse electrochemical generated substances, which alter the reduction process of the oxidised-PCT form.

log *I_pa_* (µA) = (0.61 ± 0.01) log *ν* (mV s^−1^) + (0.25 ± 0.02) (*R*^2^ = 0.99814) (3)

log *I_pc_* (µA) = (−0.35 ± 0.01) log *ν* (mV s^−1^) + (−0.56 ± 0.03) (*R*^2^ = 0.98438) (4)

The well fit of Equation (3) supports a diffusion-controlled electrode anodic reaction. The theoretical slope values of 0.5 and 1.0 for the linear relationship between log(*I_pa_*) versus log(*ν*) indicate pure diffusion control and pure adsorption control processes, respectively. Our results provide the slope value of 0.61 for the oxidation process, Equation (3), supporting an electrode reaction controlled by a diffusion process (electrochemical quasi-reversibility), although the reduction process can suffer essential differences. The slope of 0.5 indicates a reversible process at low scan rates, whilst an irreversible process at high scan rates. Nonetheless, the quasi-reversibility is more plausible for high analyte concentrations (300 μM), considering that Δ*Ep* shifts with *ν*. Comparatively, for small concentrations (<100 μM), no peak-to-peak separation occurs, and the diffusion process is no longer limiting with the electron transfer occurring via a surface-adsorbed species. This behaviour indicates that the PCT electrode reaction depends not only on the working electrode surface and material but also on the analyte concentration. This last reasoning is logical if the redox species achieved at the electrode surface changes with the analyte concentration.

We think all of the above considerations are better understood if we consider that during the PCT oxidation process, its conjugation system changes to a less stable product’s conjugation model, suggesting a complex and less robust π–π interaction between the oxidation product and the Stv particle’s surface. This mechanism facilitates the oxidation of PCT with an over-potential which decreases ~50 mV against the unmodified CPE. Further, the PCT molecule can exist in solution as four conformers with different stability, potentially changing with pH. The planar-geometry *trans*-conformers (E or Z), more easily solvated than the nonplanar-geometry *cis*-conformers (E or Z), present an intramolecular bond between a benzene hydrogen atom and the carbonyl oxygen, introducing stabilisation in the trans-conformer planarity [55,56]. The intramolecular bonding in *trans*-conformers also adds stiffness to these structures, making their folding even more difficult but facilitating solvation.

### 2.4. Effect of pH

**Figure 3:**
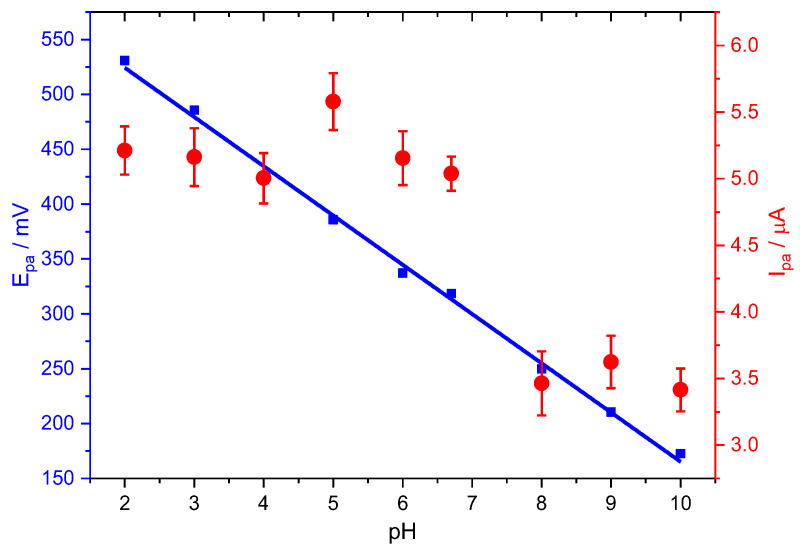
Effect of pH on the peak potential (blue) and peak current (red) for the oxidation of PCT, employing Stv-CPE, of 100 µM PCT solutions in PBS (0.1 M, pH 6.7).

Figure 3 shows the linear dependence, Equation (5), between the PCT peak potentials and the solution pH between 2.0 and 10.0 on the oxidation potential and peak current of the PCT electrochemical response at Stv-CPE by DPV.

*E_pa_* (mV) = (−44.88 ± 0.90) pH + (614.02 ± 6.00) (*R*^2^ = 0.99712) (5)

The oxidation peak potential (*E_pa_*) shifted negatively with increasing pH until the first dissociation constant (pK_a_ = 9.6), suggesting a direct involvement of protons in the analyte’s oxidation [9], probably according to the following chemical reactions:

(6) $$AH_{m}⇄A^{m-}+mH^{+}\;K_{a}=\frac{\left[A^{m-}\right]{\left[H^{+}\right]}^{m}}{\left[AH_{m}\right]}$$

$$A^{m-}\to B+ne^{-}$$

A and AH refer to the unprotonated and protonated paracetamol, while B refers to the corresponding oxidised form. Considering the total amount of analyte, $A_{T}=\left[AH_{m}\right]+\left[A^{m-}\right]$, and the equilibrium constant *K_a_*, we arrive at $\left[A^{m-}\right]=\frac{K_{d}A_{T}}{K_{a}+{\left[H^{+}\right]}^{m}}$. However, as it is usual, $\left[H^{+}\right]\gg \;K_{a}$, so we can relate the peak potential and pH as follows [57]:

(7) $$E_{P}=cte+\frac{2.303\;R\;T}{\left(n+\alpha \right)\;F}\;logK_{a}-\frac{2.303\;m\;R\;T}{\left(n+\alpha \right)\;F}\;pH$$

Equation (7) shows that with increasing pH, the peak potential for oxidation of PCT shifts to more negative potentials.

The slope of −44.88 mV pH^−1^ in Equation (5) fits well with the slope value of Equation (7) for a two-electron oxidation process. Further, it is also close to the Nernst theoretical value of 59 mV pH^−1^, suggesting a proton-to-electron ratio near unity. However, because of the difficulty of ascertaining the exact number of electrons, we can compare this slope value with the theoretical value from the Nernst equation derivative with pH, *dEp*/*dpH* = 2.303 *mRT*/(*αnF*), although valid only for reversible processes. We found an *m*/(*αn*) ratio value of 0.8 for the oxidation process, suggesting a quasi-reversible process. Note that *m* is the number of protons involved in the electrochemical reaction, *n* is the number of electrons, and α is the charge transfer coefficient.

Figure 3 also shows the pH dependence peak current of the PCT oxidation in the negative-going scan. As stated in the following equation, the peak current also lowers with pH for an irreversible process [57]:

(8) $$I_{p}\propto A_{T}\;\frac{K_{a}}{K_{a}+{\left[H^{+}\right]}^{m}}\;e^{\left(-\frac{\left(n+\alpha \right)\;F}{R\;T}\;\eta \right)}$$

However, the plot of the anodic peak intensity against pH shows a distorted line at neutral pHs, probably due to the suggested dimerisation of PCT [58], facilitating the redox process. The total concentration of the oxidable form, the protonated form of PCT (HPCT), decreases with pH, similarly to the oxidation peak current, but this does not explain the slight increase in the peak current at neutral pHs. Nonetheless, we think the hydrogen bridge-bond intermolecular dimerisation of PCT between the hydroxyl and the carbonyl groups, typical of the carboxylic acids, or the topological changes in the PCT conformers could be compatible with the peak intensity changes at neutral pHs [7,59]. Subsequently, the peak current decreases with pH, even at alkaline pH, because the total concentration of the PCT oxidable form also keeps decreasing.

### 2.5. Electrochemical Kinetic Parameters

To evaluate the redox mechanism of an electrochemical process, we need to know several kinetic parameters, particularly the heterogeneous rate constant *k*^0^, the electron transfer coefficient for the cathodic α and anodic *β* processes, and the mass transfer rate *m_T_*. However, the electrochemical process often involves multiple electron-transfer reactions with *n* = two or more electrons. It shows a single voltammetric wave, seemingly a single step, as in Figure 2. This behaviour usually appears in many molecules with a delocalised electrons system, even undergoing a two-electron process. The second process occurs quickly, typically due to changes in the molecular structure, simultaneously with or after the first process, or destabilisation of intermediates by changes in solvation [60]. Therefore, in this work, the second oxidation of PCT would occur at a lower positive potential than that required for the first oxidation process, overlapping both peaks, which is a condition to see only one anodic peak [60].

Even though theoretically possible, the transfer of two or more electrons occurs sequentially, one at a time, with each reaction having characteristic parameters. Nonetheless, it is adequate to use an overall stoichiometry reaction for simplification. In these cases, all of the kinetic parameters calculated take apparent values (overall values), averaging in some way the values from all reactions involved and the possible whole effect of the operational mode (solvent type, scan rate, electrode material, and others) [61,62]. This way, the calculated parameters have a different significance than the corresponding parameters of a single-electron transfer.

The study of the CV waves allows us to derive valuable kinetic parameters, which are helpful for better understanding the redox process. The transfer coefficient *α* or *β* affects the CV wave symmetry, showing cathodic peaks more rounded than anodic peaks for transfer coefficient values <0.5 and lower peak heights. However, the converse holds for values >0.5, although changes in the transfer coefficient do not usually affect the peak potential difference [61]. For quasi-reversible electron transfers, the wave’s shape mainly depends upon Δ*E_p_* and is not very dependent upon the transfer coefficient, which cannot be accurately determined unless we use scan rates large enough to make the wave irreversible [63].

In this work, the peak-to-peak separation is scan rate-dependent, suggesting kinetic complications. Therefore, deciding whether this wave features a one-electron or a two-electron process is impossible. For this reason, we cannot accurately calculate the number of electrons, *n*, exchanged. However, we can arrive at an average value of the α*n* or β*n* product for diffusion control systems, as is our case, from the slope of the plot of *E_p_* versus ln(*ν*), according to the following equations [64]:

(9) $$E_{pa}=K-\left[\frac{R\;T}{\beta \;n\;F}\right]\;Ln\;\nu$$

(10) $$E_{pc}=K-\left[\frac{R\;T}{\alpha \;n\;F}\right]\;Ln\;\nu$$

The average value for the charge-transfer coefficient of the anodic process derives from the slope $\left[\frac{RT}{\beta nF}\right]$ of Equation (9) (slope = 0.01448) $\left(\bar{\beta }n_{a}=2.36\right)$. The plot of Equation (10) shows a poor fit, which means that the transfer coefficient α changes strongly with the scan rates. Even though the mechanistic electrochemical oxidation of PCT is definitively unclear, the most accepted value for the number of electrons exchanged *n* is 2 and, consequently, β would take the value near unity, suggesting that the rate-determining step is the first electron transfer.

Based on Laviron’s theory [65], we can determine the heterogeneous rate constant *k*^0^ by measuring the variation in peak potential with the scan rate. The standard (or conditional) rate constant *k*^0^ is an essential parameter in electrode kinetics, measuring the kinetic facility of the redox couple and relating the speed of electron transfer between an electroactive species and the electrode surface. Thus, the larger the value of *k*^0^, the faster equilibrium is attained. The standard heterogeneous rate constant has units of cm s^−1^, which result from the concentration of redox-active species in mol cm^−3^ and electron transfer to an electrode area in cm^2^. We can calculate *k*^0^ (cm s^−1^) as a function of *ν* and Δ*E_p_* through the following equation for an irreversible surface-bound species [64],

(11) $$E_{p}=E^{0}-\frac{R\;T}{\alpha \;n\;F}\;Ln\left(\frac{\alpha \;F\;\nu }{R\;T\;k^{0}}\right)$$

However, the above equation fits another empirical equation derived from the bibliography [24]:

(12) $$∆E_{p}=201.39\;\log\frac{\nu }{k^{0}}-301.78$$

(13) $$\log k^{0}=\log\nu -\frac{∆E_{p}+301.78}{201.39}$$

The *k*^0^ values obtained from the different scan rates in V are in Table 3.

On the other hand, we can alternatively elucidate the reversibility or irreversibility of an electrochemical system by comparing the value of *k*^0^ with the rate of mass transport, *m_T_*, which depends on the scan rate. According to the Einstein equation, we can relate the mean diffusion layer thickness with the potential window and the scan rate according to the following equation [57]:

(14) $$\delta =\sqrt{\pi D\frac{∆E_{p}}{\nu }}$$

Further, the diffusion layer also relates to the rate of mass transport:

(15) $$m_{T}=\frac{D}{\delta }\;$$

The *m_T_* values (Table 3) grow with the scan rate because the faster the voltage scan rate, the thinner the diffusion layer, encouraging greater electrochemical irreversibility. We found comparable values for *m_T_* and *k*^0^ (Table 3), which suggest a predominant diffusion process and some irreversibility in electrode processes. Note that if *k*^0^ << *m_T_*, the process is irreversible and reversible for *k*^0^ >> *m_T_*.

Another way to evaluate the electrochemical reversibility is through the parameter Ψ [61], related to the peak-to-peak separation potential according to Equation (16) and also used as an electrochemical reversibility parameter:

(16) $$\psi =\frac{-0.6288+0.0021{\;∆E}_{p}}{1-0.017\;{∆E}_{p}}$$

The parameter Ψ also allows, for comparison purposes, re-calculating the heterogeneous rate constant value, *k*^0^, according to Equation (17), knowing the rest of the parameters:

(17) $$k^{0}=\psi \sqrt{\frac{\pi \;D\;n\;F\;\nu }{R\;T}}$$

The parameter Ψ is the ratio of the rate of charge transfer *k*^0^ to the mass transfer, represented by the denominator, and its calculation allows us to address the electrochemical reversibility. In Equation (17), *D* = 0.664 × 10^−5^ cm^2^ s^−1^ is the diffusion coefficient of the electroactive species taken from the bibliography [66], *n* is the number of electrons transferred in the electrochemical reaction, *F* is the Faraday constant (96,500 C mol^−1^), *R* is the molar gas constant (8.31 J K^−1^ mol^−1^), and *T* is the absolute temperature.

Calculating the upper limit of the heterogeneous rate constant determined by the Ψ method contributes to various factors. Thus, the uncompensated ohmic potential loss is an error source in the peak potential difference. Peak currents become pretty significant for the rapid scan rates required to study fast electrode reactions, and even relatively small solution resistances can introduce serious errors. Moreover, the effects of uncompensated *iR* dropping qualitatively are very similar to kinetic effects [61]. Further, according to Equation (16), the parameter *Ψ* is only adequate for a particular range of the peak-to-peak potential difference because the number of electrons involved in the redox process also affects it. Thus, considering that the lower the value of *Ψ*, the greater the Δ*E_p_* separation for a multi-electron process, Equation (16) is unusable for high scan rates generating an irreversible process.

The parameter *Ψ* values obtained from Equation (16) (Table 3) were between 0.1 and 1.5, corresponding to a quasi-reversible/irreversible system [67,68,69]. The parameters used in Equation (17) were in mV s^−1^, Δ*E_p_* in mV, *n* = 2 electrons, and *D* = 0.664 × 10^−5^ cm^2^ s^−1^ taken from the bibliography [70]. The values found for *k*^0^ from Equation (17) are in Table 3. Reversibility or irreversibility is a function of voltage scan rate switching over for *Ψ* ~ 1. The significant difference between a reversible and an irreversible voltammogram is the potential separation between the anodic and cathodic peaks, which relates to *Ψ* and *k*^0^. For *Ψ* values closing to zero, the case approaches for irreversible electron transfer [61]. For this reason, alternative equations are helpful to evaluate the standard rate constant from CV curves using the peak-to-peak separation Δ*E_p_* of the anodic and cathodic waves when the Δ*E_p_* values measured lie outside the range of *Ψ* values of Nicholson’s treatment [71].

A direct proportionality exists between *i*_0_ and *k*^0^, and the concentration of the electroactive species *C*, through Equation (18), decaying exponentially with the free activation energy for the charge transfer process at equilibrium [69,72]:

(18) $$i_{0}=n\;F\;k^{0}\;C$$

The corresponding values found for *i*_0_ (current density), shown in Table 3, agree with the experimental values provided by the instrument once considered the effective electrode surface. Alternatively, the exchange-current density, or current density flowing equally in both directions at equilibrium or in the absence of net electrolysis and at zero overpotential, is frequently used as an essential kinetic parameter in place of *k*^0^. Ideally, the current density would be as high as possible to achieve fast kinetics.

The Randles−Sevcik equation [73,74] relates the peak current *I_p_* with the scan rate and other electrode parameters according to the following equation:

(19) $$I_{p}=0.446nFAC{\left(\frac{nFvD}{RT}\right)}^{1/2}$$

The parameter *A* (cm^2^) is the electrochemically efficient electrode surface area (usually treated as the geometric surface area). The other parameters take the same meaning as previously defined. The Randles−Sevcik equation for reversible electrochemical processes may calculate diffusion coefficients and assess whether an analyte remains homogeneously in solution (involving diffusing redox species freely) before analysing its reactivity. However, this equation allows diverse calculations; taking the value of 0.664 × 10^−5^ cm^2^ s^−1^ for *D*, the diffusion coefficient of PCT [70], we can calculate *A* for each scan rate used [75], finding the values included in Table 3 for the oxidation processes.

Notwithstanding, only for comparison purposes, we re-calculate the diffusion coefficient *D* for the PCT through the Randles−Sevcik or Berzins–Dalahay equation [64]. In Equation (19), we used an electrode area *A* = 0.5 cm^2^, PCT concentration *C*_0_ = 0.3 mM and *n* = 2, finding for *D* the values included in Table S3 for the oxidation and reduction processes, which are of the same order of magnitude as the values provided in the bibliography.

According to the above calculations, the Stv-CPE electrode has good electronic transport properties that produce quasi-reversible processes. The good electrochemical activity for the PCT redox reaction is probably the result of the favourable π–π and π-n interaction formed between the PCT molecules and the π system and lone pair electrons of the Stv material. The lone pairs on the hydroxyl oxygen and the nitrogen atom of the amide group (acetamide), joined to the carbonyl oxygen and the benzene π cloud, generate a planar PCT molecule with an extensively conjugated system. This conjugated system shows more reactivity than usual due to two *o-* and *p-*directing electron-donating groups in resonance with the benzene aromatic ring, thus facilitating π–π interactions. The conjugation process significantly reduces the basicity of the hydroxyl oxygen and amide nitrogen, increasing the hydroxyl acidity.

Consequently, PCT is neutral at pH 6.7. The amide group generally acts as a hydrogen bond donor, whereas the carbonyl group acts as a hydrogen bond acceptor. However, the hydroxyl group can act as a donor and acceptor. These possibilities facilitate the formation of intra- and inter-molecular hydrogen bridges, plausibly with the OH groups in Stv. Protonated PCT in the NH_2_^+^ group only exists at very acidic pHs (<0.14), theoretically coexisting with the neutral form. However, protonation in the C=O group is less plausible because the O atom is part of a conjugated system. On the other hand, very low pHs facilitate the better formation of dimers (poly-condensation process).

### 2.6. Effect of PCT Concentration and Calibration Plot

**Figure 4:**
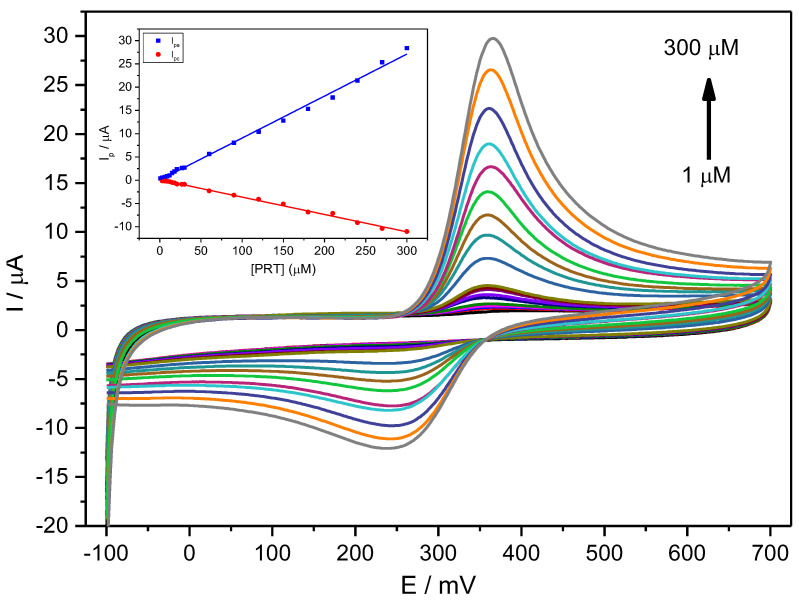
CV voltammograms for a PCT concentration range of 1–300 µM (1.0, 3.0, 6.0, 9.0, 12, 15, 18, 21, 27, 30, 60, 90, 120, 150, 180, 210, 240, 270, 300 µM) in PBS (0.1 M, pH 6.7) employing the Stv-CPE and *υ* = 100 mV s^−1^. The inset represents the plots of the *I_p_* for anodic (blue) and cathodic (red) peaks vs. the PCT concentration (1–300 µM).

Figure 4 shows CV voltammograms for a narrow range of PCT concentrations, indicating a quasi-reversible behaviour with relatively weak redox current peaks at 0.33 V and a constant Δ*E_p_* of about 0.059 V (Figure 4).

For a fixed scan rate, the intensity of the anodic and cathodic peaks shows a direct and well-correlated relationship with the analyte concentration between 1 μM and 300 μM (Figure 4, Inset) and Equation (20):

*I_pa_* (µA) = (0.090 ± 0.001) [PCT] (µM) + (0.033 ± 0.002) (*R*^2^ = 0.99560) (20)

*I_pc_* (µA) = (0.037 ± 0.001) [PCT] (µM) + (0.092 ± 0.003) (*R*^2^ = 0.99575) (21)

The ratio of the slopes from the anodic current against the cathodic current differs from unity (equal to ~2), indicating a specific chemical or electrochemical irreversibility. This irreversibility means that the analyte diffusion coefficient in solution differs for the reduced and oxidised forms. The anodic peak shows a higher intensity than the cathodic peak, consequently providing an improved sensitivity for analytical purposes.

However, we used DPV as a sensitive electroanalytical technique to determine low PCT concentrations in PBS at the optimised scan rate of 10 mV s^−1^. Under optimum conditions, the variation in PCT’s oxidation peak current (*I_pa_*) with the PCT concentration was linear in the range from 0.6 μM to 100 μM, according to the following equation (Figure S10):

*I_p_* (µA) = (0.05 ± 0.00) [PCT] (µM) + (0.27 ± 0.04) (*R*^2^ = 0.99351) (22)

The sensitivity of the method was 20.0 μA μM^−1^ in the concentration range between 0.6 μM and 100 μM, whilst the best detection (LOD) and quantification (LOQ) limits were 0.2 μM and 0.5 μM, respectively. We calculated the LOD and LOQ from the calibration lines as *kSD/b*, where: *k* = 3 for LOD, *k* = 10 for LOQ, with *SD* the standard deviation of the intercept, and *b* the slope of the calibration line.

We did not find a decrease in the anodic peak intensity at high concentrations, contrarily to other authors [58], probably because the paracetamol species remain unchanged, without forming an intermolecular bridge, at the concentrations used in this work. Further, the LOD values found were similar to those previously provided by other electrodes based on clay minerals, although much better than those based on oxide ceramics (Table 4, Figure S11).

### 2.7. Validation and Practical Analytical Applications

**Figure 5:**
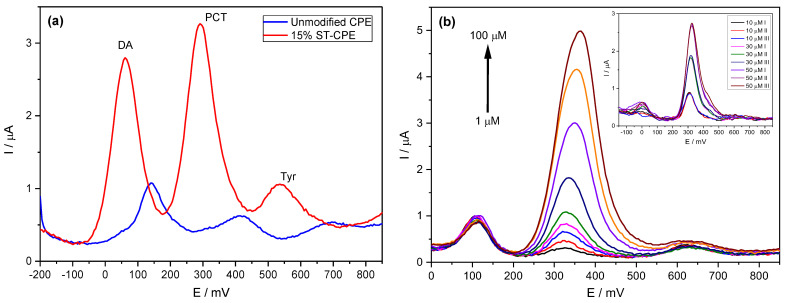
(**a**) DPV of 50 µM PCT in PBS (0.1 M, pH 6.7) in the presence of 50 µM dopamine (DA) and 50 µM of tyrosine (Tyr). (**b**) DPV of PCT (1–100 µM) using the Stv-CPE in the presence of 10 µM of DA and 50 µM of Tyr in PBS (0.1 M, pH 6.7). (The inset corresponds to three spiked replicate PCT serum samples).

We previously analysed standard solutions containing PCT mixed with DA and Tyr to determine the possible interfering effect of compounds similar to PCT and frequently found in similar matrices. The determination of PCT using the prepared CPE was possible even in the presence of DA and Tyr, with two entirely resolved peaks on Stv-CPE for these three species (Figure 5a). However, varying the PCT concentration in the presence of fixed DA and Tyr concentrations, the peak profiles of the PCT found by DPV were similar to those achieved using standard analyte solutions (Figure 5b).

The calibration line working with complex samples shows a slope (sensitivity) similar to that obtained with high concentrations of analyte standards. The analytical sensitivity depends strongly on the electrode composition (Table 4), with better sensitivity for the electrodes, including clay minerals in their preparation. Nonetheless, a deeper comparison between all analytical parameters requires considering a more accurate similarity in the material granulometry.

We evaluated the repeatability and reproducibility of the developed procedure using DPV in determining PCT 50 µM in PBS solutions (pH 6.7), finding RSD values around 4.3% and 4.8%, respectively. Further, the developed electrode shows high stability, and depending on its size/length (see Section 3.5), up to 15 measurements are even possible with analytical changes below 5%.

Furthermore, we evaluated the applicability of the developed electrode, Stv-CPE, to the pharmaceutical samples. For this purpose, we prepared various PCT solutions using two commercial solid formulations, Parantal and Doliprane, bought in a local pharmacy. Each tablet of Parantal and Doliprane contained 500 mg and 1000 mg of PCT per g tablet, respectively. We added various amounts of PCT to each sample solution at 0.1 M PBS at pH 6.7, diluting them to lie within the range of the calibration plot and recording them under optimum conditions using an external calibration procedure. At room temperature, the concentrations obtained are listed in Table 5. Table 5 shows recoveries of 98.15%, which relates to the sample’s nature and the elements’ concentration levels [76,77], indicating an acceptable validity of the proposed procedure to determine PCT in complex matrices. Conclusively, the developed electrode is reliable for application in a pharmaceutical environment. The method’s precision (% RSD) was less than 4.0%, while other analytical parameters were as listed in Table 5 for comparison with the related published papers.

The analysis of the serum samples (Figure 5b, inset) showed two additional peaks due to uric acid and xanthan, already detected in our previous works [78,79]. However, their electrochemical peaks appeared out of the potential range corresponding to the PCT. Further, the repeatability of the procedure showed high precision, as reported in Figure 5b (inset).

## 3. Materials and Methods

### 3.1. Reagents

Paracetamol, dopamine, tyrosine, and potassium ferricyanide were from Sigma-Aldrich (Saint Louis, MO, USA), and HCl, NaOH, KH_2_PO_4_, K_2_HPO_4_, paraffin oil and PVC capillary tubes (i.d. 3 mm) were from Honeywell Fluka, Fisher Scientific (Madrid, Spain). Graphite powder (23 micros, 99.95) was from Pro-GraphiteShop (Untergriesbach, Germany), and Rhassoul Clay was from Tamdalet in Middle Atlas Mountains (Fez-Boulmane area, Morocco). The serum samples with serum group (A) rhesus negative were provided from the blood transfusion centre (Tetouan, Morocco), and the commercial solid formulations, Parantal and Doliprane, were bought from a local pharmacy. KH_2_PO_4_ and K_2_HPO_4_ were used to prepare a buffer solution (PBS) at pH 6.7. All experiments were conducted at room temperature, and all reagents were of the highest analytical degree.

### 3.2. Apparatus

A Potentiostat/Galvanostat/ZRA type Interface 1010 T (Gamry Instruments, PA, USA) controlled by a computer for voltammetric measurements and a VoltaLab PGZ301 (Radiometer Analytical SAS, Vileurbanne, France) for electrochemical impedance spectrometry (EIS) were employed. The transformation of the chemical data into electrical signals was ensured through Gamry Framework and VoltaMaster software. The three-electrode single-compartment cell contained a reference electrode Ag/AgCl (in saturated KCl solution), a platinum wire as the counter electrode and the modified electrode Stv-CPE as a working electrode. Cyclic voltammetry (CV), differential pulse voltammetry (DPV) and electrochemical impedance spectroscopy were used as analytical techniques.

The Stv clay material was characterised by a scanning electron microscope (SEM) model JSM-6480LV (JEOL Ltd., Akishima, Tokyo, Japan) coupled to an energy-dispersive X-ray spectrometer system (EDS) Oxford model D6679 (Oxford Instruments, Abingdon, UK), operated 20 kV acceleration voltage and 5 nA probe current. We obtained the corresponding EDS spectra, scanning each sample for 200 s with a 10 mm working distance. The Epsilon 1 X-ray fluorescence (XRF) spectrometer (Malvern Panalytical, Malvern, UK) was used to analyse the clay composition. The particle size was measured by transmission electron microscopy (TEM) using a JEOL 1010 electron microscope (JEOL Ltd., Akishima, Tokyo, Japan) operating at 90 kV. For observation in TEM, the samples were put directly onto a 200-mesh (3 mm diameter) copper grid coated with a holey carbon film and transferred to a TEM load lock. Recording of infrared spectra was obtained by a Fourier transform infrared spectroscope (FTIR) JASCO model 4700 (JASCO Analytica Spain, Madrid, Spain), with a single-reflection ATR accessory incorporating a monolithic diamond crystal ATR PRO ONE (Laser Components, NH, USA). All spectra were over the wavelength range of 4000–650 cm^−1^ at a resolution of 4 cm^−1^, averaging 100 scans, and based-corrected transformed later.

### 3.3. Preparation of Carbon Paste Electrodes

The Stv-CPE was elaborated by thoroughly hand mixing 1 g of graphite powder, 150 mg of Stv clay, and an appropriate amount of mineral oil (300 μL g^−1^). The mixture was added to an agate mortar and homogenised by a pestle for approximately 30 min. The homogeneous mixture was inserted in a 3 mm diameter cylindrical plastic tube, fixing a copper wire to establish electrical contact with the external circuit. The electrode surface was rinsed with ethanol and distilled water before use. The electrodes were polished with No. 1200 emery paper to remove extra composite material and wiped gently with weighing paper. The CPE was prepared similarly to the Stv-CPE preparation except for the absence of Stv in the paste. Finally, the electrode surface was cleaned electrochemically by applying several cyclic voltammograms. After each test, we cleaned the electrode surfaces again to remove traces of PCT remaining on the surface.

### 3.4. Real Sample Preparation

To evaluate the applicability of the Stv-CPE, we prepared two real samples (Parantal and Doliprane tablets), containing 500 mg of PCT (Parantal, Laprophan Laboratoire, Casablanca, Morocco) and 1000 mg of PCT (Doliprane, Sanofi-Aventis, Gentilly, France) and spiking growing amounts of PCT to each sample solution in 0.1 M PBS at pH 6.7.

The biological medium was prepared by mixing 4 mL of serum with 21 mL of PBS (0.1 M, pH 6.7) and shaking for 30 min. The PCT additions were performed identically as pharmaceutical samples.

### 3.5. Standard Procedure of Measurements

After optimising the working solution, we employed a classic three-electrode cell for electrochemical measurements of the sample in 25 mL 0.1 M PBS solution at pH 6.7 using variable potential intervals: for CV, the potential range was between −0.1 and 0.7 V, while for DPV, it varied between −0.2 and 0.9 V.

The CV measurements started at the lowest studied potential and progressed in the anodic direction until reaching the highest potential. Then, they progressed in the cathodic direction to complete the cycle. Regarding the DPV measurements, they were conducted in the anodic direction with a pulse size of 25 mV and a sweep rate of 10 mV s^−1^ after an optimised accumulation time of 4 min. This sweep gave us better resolution and a better signal-to-noise ratio.

After each voltammogram, we removed the outer part of the electrode with a cutter, rinsed with PBS solution and stored it at ≈25 °C. Subsequently, the electrode was polished using the method discussed in Section 3.3 to prepare it for the following analysis. The storage stability of the sensor was within 5.0% for up to 15 measurements.

## 4. Conclusions

In this work, we successfully evaluated a CPE modified by the Stv mineral to determine PCT in complex matrices. CV and DPV were excellent techniques to improve the electrochemistry of PCT using an Stv-CPE. The linear dynamic range for PCT determination was between 0.6 and 100.0 µM. The LOD was 0.2 µM, with the best analytical sensitivity of the calibration plot of 20.0 μA μM^−1^, greatly improved by using an accumulation time of 4 min before analysis. The accuracy of the Stv-CPE indicated excellent suitability for paracetamol’s selective determination in complex matrices from pharmaceutical and biological samples. The prepared electrode’s advantages include the possibility of working under mild chemical conditions in any general laboratory.

## Figures and Tables

**Table 1:** Several characteristic potentials as a function of the scan rate.

*ν*, V s^−1^	*E_pa_*, V	*E_pc_*, V	*E_a(_*_1/2)_, V	*E_a_^0^* or *E_a_*_(*i*)_, V	*E_a_*_(_*_e_*_)_, V
0.025	0.346	0.268	0.307	0.308	0.275
0.050	0.352	0.263	0.307	0.312	0.276
0.075	0.357	0.257	0.307	0.314	0.278
0.100	0.360	0.253	0.306	0.320	0.281
0.125	0.365	0.250	0.307	0.324	0.283
0.150	0.364	0.246	0.305	0.326	0.286
0.175	0.370	0.239	0.304	0.328	0.286
0.200	0.371	0.235	0.303	0.330	0.285
0.225	0.375	0.229	0.302	0.334	0.289
0.250	0.377	0.225	0.301	0.336	0.289
0.275	0.380	0.220	0.300	0.338	0.288
0.300	0.381	0.220	0.301	0.340	0.289

**Table 2:** Peak potentials for diverse types of electrodes vs. Ag/AgCl.

*E_pa_*, V	*E_pc_*, V	Δ*E_p_*, V (Ag/AgCl)	Electrode Type	Reference
0.368	0.101	0.267	Graphene	[13]
0.458	0.088	0.370	Fe_2_O_3_	[20]
0.340	0.240	0.100	TiO_2_	[22]
0.345	0.174	0.171 (SCE)	ZrO_2_	[24]
0.240			Cu(II)-doped zeolite	[30]
0.310	0.240	0.070	Stevensite	This work

**Table 3:** Values of *k*^0^ for PCT at different scan rates.

*υ,* mV s^−1^	*k*^0^, cm s^−1^ (Equation (13))	*m_T_*, cm s^−1^ (Equation (15))	*ψ* (Equation (16))	*k*^0^, cm s^−1^ (Equation (17))	*i*_0_, A cm^−2^ (Equation (18))	*I_pa_*, A (Theor.)	*A_a_*, cm^2^ (Equation (19))
25	3.44 × 10^−4^	8.25 × 10^−4^	1.46 × 10^0^	6.12 × 10^−3^	1.99 × 10^−5^	2.39 × 10^−6^	1.20 × 10^−1^
50	6.03 × 10^−4^	1.09 × 10^−3^	8.47 × 10^−1^	5.04 × 10^−3^	3.49 × 10^−5^	4.73 × 10^−6^	1.35 × 10^−1^
75	8.03 × 10^−4^	1.26 × 10^−3^	5.98 × 10^−1^	4.36 × 10^−3^	4.65 × 10^−5^	6.54 × 10^−6^	1.41 × 10^−1^
100	9.84 × 10^−4^	1.40 × 10^−3^	4.88 × 10^−1^	4.10 × 10^−3^	5.70 × 10^−5^	8.25 × 10^−6^	1.45 × 10^−1^
125	1.13 × 10^−3^	1.52 × 10^−3^	4.05 × 10^−1^	3.81 × 10^−3^	6.53 × 10^−5^	9.22 × 10^−6^	1.41 × 10^−1^
150	1.31 × 10^−3^	1.64 × 10^−3^	3.77 × 10^−1^	3.88 × 10^−3^	7.57 × 10^−5^	1.11 × 10^−5^	1.47 × 10^−1^
175	1.32 × 10^−3^	1.68 × 10^−3^	2.88 × 10^−1^	3.20 × 10^−3^	7.64 × 10^−5^	1.15 × 10^−5^	1.50 × 10^−1^
200	1.44 × 10^−3^	1.77 × 10^−3^	2.65 × 10^−1^	3.15 × 10^−3^	8.31 × 10^−5^	1.31 × 10^−5^	1.57 × 10^−1^
225	1.43 × 10^−3^	1.80 × 10^−3^	2.17 × 10^−1^	2.73 × 10^−3^	8.28 × 10^−5^	1.25 × 10^−5^	1.51 × 10^−1^
250	1.49 × 10^−3^	1.87 × 10^−3^	1.96 × 10^−1^	2.60 × 10^−3^	8.62 × 10^−5^	1.35 × 10^−5^	1.56 × 10^−1^
275	1.49 × 10^−3^	1.91 × 10^−3^	1.70 × 10^−1^	2.37 × 10^−3^	8.66 × 10^−5^	1.37 × 10^−5^	1.59 × 10^−1^
300	1.60 × 10^−3^	1.98 × 10^−3^	1.65 × 10^−1^	2.40 × 10^−3^	9.25 × 10^−5^	1.49 × 10^−5^	1.61 × 10^−1^

**Table 4:** Comparison of the limits of detection and quantitation (if provided) found in the literature related to other works using related electrode types. The first three rows correspond to HPLC methods.

Electrode Type	LOD	LOQ	Ref.
HPLC-PDA (FTIR-derivative)	13.0 μM (137.5 μM)	27.7 μM (458.3 μM)	[5]
HPLC-PDA	2.05 μM	6.28 μM	[6]
RP-HPLC-PDA (RP-HPLC-Fl)	0.2 μg mL^−1^ (0.1 μg mL^−1^)	0.8 μg mL^−1^	[7]
Copper zinc ferrite NPs/CPE	0.0885 μM		[10]
Graphene/GCE	0.032 μM		[13]
Pyrolytic carbon films	1.4 μM		[15]
GO/poly (Val)/CPE	0.29 μM	0.96 μM	[16]
Poly (3, 4-ethylene dioxythiophene)/GCE	0.40 μM		[17]
Nevirapine/CPE	0.77 μM		[18]
CdO/CPE	0.07 μM	0.1 μM	[19]
Fe_2_O_3_/CPE	1.16 μM		[20]
TiO_2_-WO_3_/CPE	10.18 nM	34.32 nM	[21]
TiO_2_/CPE	5.2 µM	18 µM	[22]
ZnO/functionalise MWCNT/CPE	0.23 μM	0.79 μM	[23]
Zirconium Oxide/CPE	0.68 μM		[24]
MgO/CPE	6.2 μM	20.9 μM	[25]
Bismuth oxide and oxynitrate heterostructures/SPE	3.64 μM		[26]
SnO_2_/CuS, SnO_2_/SnS, Cu@SnO_2_/SnS/CPE	0.06 μM		[29]
Copper(II) doped zeolite/CPE	0.1 μM		[30]
Zeolite/CPE	0.04 μM		[31]
Safranin/CPE	0.47 μM		[32]
Clay/CPE	0.00527 μM		[33]
Nano-sepiolite clay-multiwall carbon nanotubes (Mg_4_Si_6_O_15_(OH)_2_·6H_2_O)-(MWCNT)/CPE	0.018 μM		[34]
Clay/CPE	0.14 μM	0.47 μM	[35]
Clay/CPE	0.0104 μM		[36]
Nano-clay/CPE	3.71 μM		[38]
Stevensite/CPE	0.2 μM	0.5 μM	This work

CPE: Carbon Paste Electrode; GCE: Glassy Carbon Electrode; SPE: Screen Printed Electrode.

**Table 5:** Determination of PCT in pharmaceutical formulations and serum samples.

Samples	*C_d_* (PCT), μM	*C_a_* (PCT), μM	*C_f_* (PCT), μM	Recovery [%]
**Pharmaceutical formulation Parantal (500 mg per tablet)**	10	0	8.4 ± 0.8	84.4
		5	16.0 ± 0.8	106.7
		10	20.6 ± 0.9	103.0
	25	0	24.8 ± 0.9	99.3
		10	35.5 ± 0.3	101.4
		25	47.0 ± 0.7	94.1
**Pharmaceutical formulation Doliprane (1000 mg per tablet)**	10	0	11.0 ± 0.4	110.1
		5	13.9 ± 0.9	92.4
		10	17.5 ± 0.5	87.6
	25	0	25.8 ± 0.2	103.2
		10	37.2 ± 0.3	106.4
		25	50.6 ± 0.6	101.3
**Serum samples**	-	0	0	-
		10	8.5 ± 0.0	85.3
		30	27.9 ± 0.0	93.0
		50	44.8 ± 0.0	89.5

*C_d_*: concentration of paracetamol per tablet solution, *C_a_*: concentration of purified paracetamol added, *C_f_*: concentration of purified paracetamol found.

## Data Availability

Additional results can be requested via e-mail from the corresponding authors.

## Supplementary Materials

The following supporting information can be downloaded at: https://www.mdpi.com/article/10.3390/ijms241411269/s1.
