# Supplementary material for: Electrochemical Kinetics and Detection of Paracetamol by Stevensite-Modified Carbon Paste Electrode in Biological Fluids and Pharmaceutical Formulations
Source: Int J Mol Sci. 2023 Jul 10;24(14):11269. doi: 10.3390/ijms241411269 (PMC10378910; doi:10.3390/ijms241411269)
Supplement: Supplementary file 1 [file ijms-24-11269-s001.zip › ijms-2469102-SI.pdf]

International Journal of *Molecular Sciences*

SUPPLEMENTARY MATERIALS

MDPI

Article

Electrochemical kinetics and detection of paracetamol by Stevensite-modified carbon paste electrode in biological fluids and pharmaceutical formulations

Moaad Gharous [1,2], Loubna Bounab [2], Fernando J. Pereira [3,*], Mohamed Choukairi [1,*], Roberto López [3] and A. Javier Aller [3]

[1] Laboratory of Materials and Interfacial Systems, Faculty of Science, Abdelmalek Essaadi University, BP 2121, 93002 Tetouan, Morocco.
[2] Research Group of Advanced Materials, Structures and Civil Engineering, National School of Applied Sciences of Tetouan, Abdelmalek Essaadi University, BP 2121, 93002 Tetouan, Morocco.
[3] Department of Applied Chemistry and Physics; Faculty of Biological and Environmental Sciences; Campus de Vegazana, s/n; University of León, E-24071 León, Spain.
* Correspondence: (FJP): fjperg@unileon.es; (MC): mchoukairi@uae.ac.ma

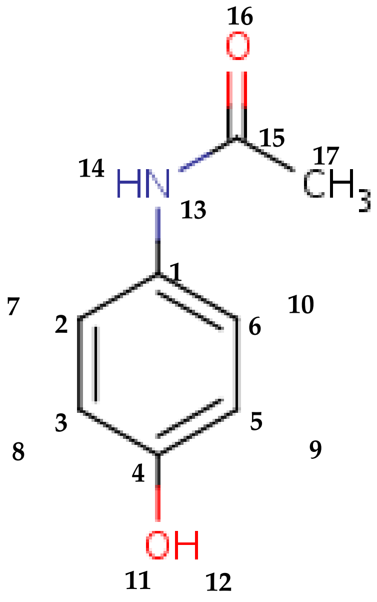

Chemical structure and numeration of the paracetamol molecule.

Table S1. Composition of the Stv clay used in this work from XRF spectroscopy.

Composition	SiO_2	Al_2O_3	TiO_2	CaO	K_2O	Na_2O	P_2O_5	Cr_2O_3	SrO	SO_3	Fe_2O_3	MnO	MgO
Mass %	69.048 ± 0.203	4.656 ± 0.033	0.397 ± 0.002	1.360 ± 0.006	1.291 ± 0.006	0.216 ± 0.053	0.573 ± 0.004	0.014 ± 0.0001	0.191 ± 0.0005	0.098 ± 0.002	3.251 ± 0.0096	0.026 ± 0.0004	18.878 ± 0.226

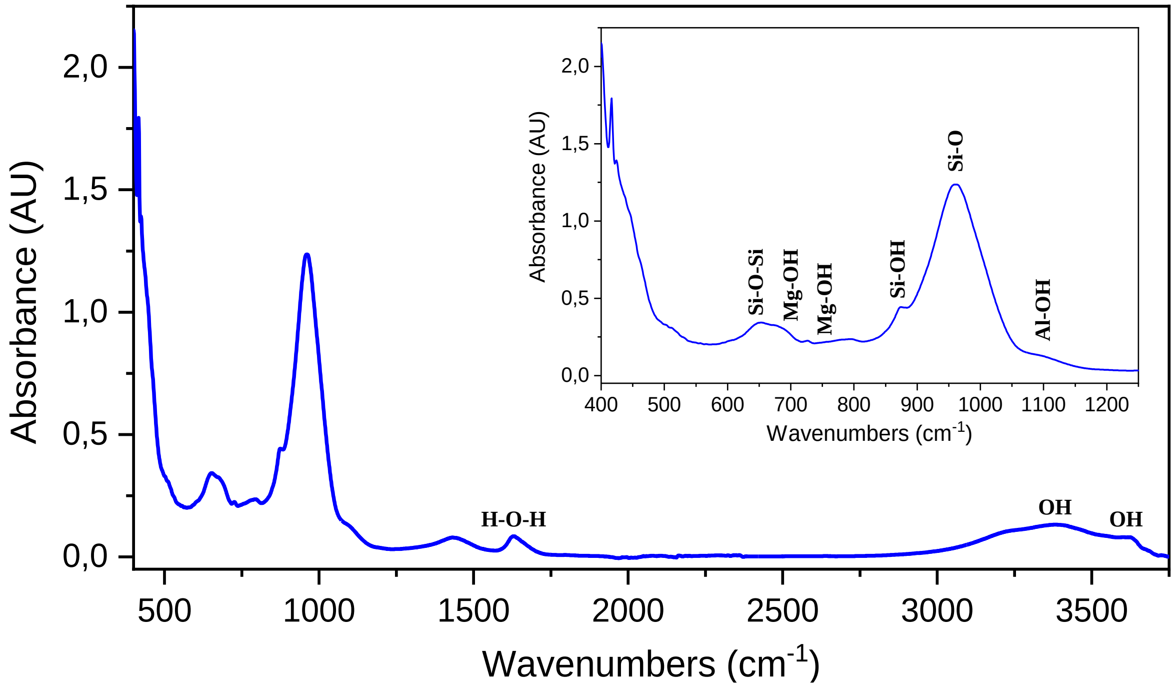

Fig. S1. FT-IR spectrum of Rhassoul clay mineral.

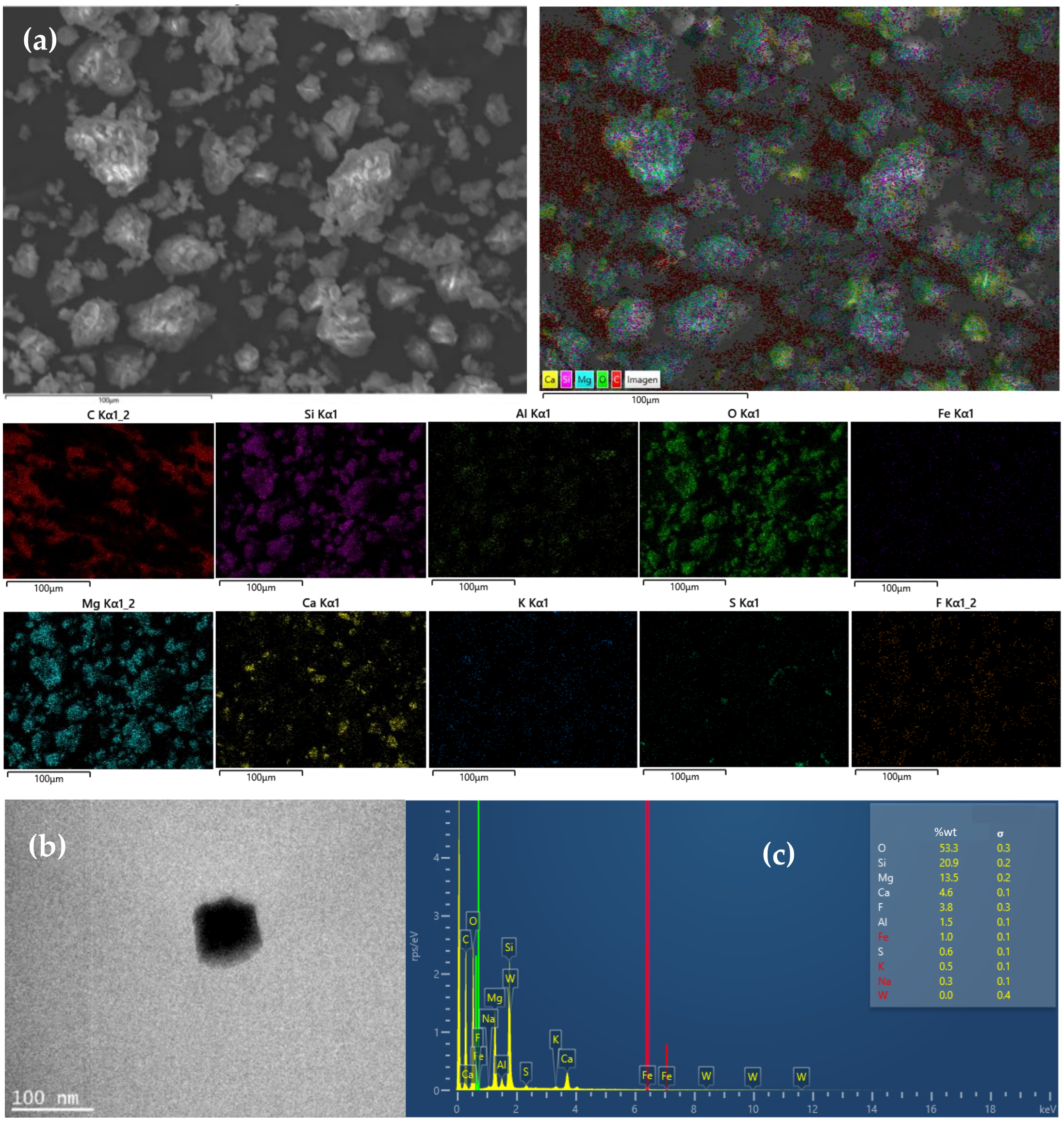

Fig. S2. (a) SEM micrograph showing the distribution of several elements. **(b)** EDS spectrum of the Rhassoul clay mineral. **(c)** TEM micrograph of the Rhassoul clay mineral.

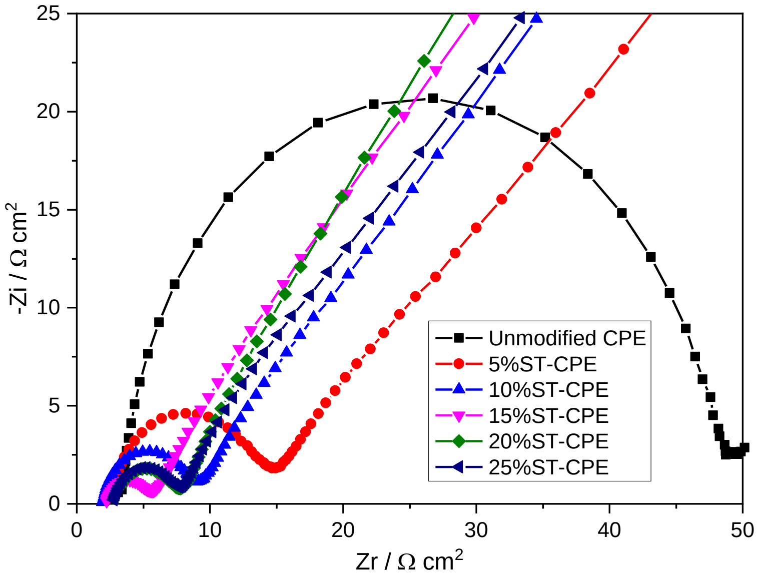

Fig. S3. Nyquist diagram of unmodified CPE and Stv-CPE for the oxidation of 0.01 M $[Fe(CN)_6]^{3-/4-}$in 0.5 M KCl.

Table S2. Experimental values of load transfer resistance and capacitance of unmodified CPE and (0-25%) Stv-CPE for the oxidation of ferro-ferri as a standard material.

Samples	R_e / Ω cm²	R_{ct} / Ω cm²	Q_{dl} / μF s^{n-1}	n
Unmodified CPE	3.345	45.03	06.68	0.947 9
05%	2.459	11.89	44.89	0.847 5
10%	1.936	06.94	59.23	0.846 1
**15%**	**2.166**	**03.40**	**129.8**	**0.792 5**
20%	2.715	04.93	105.8	0.818 2
25%	2.769	04.89	127.2	0.801 8

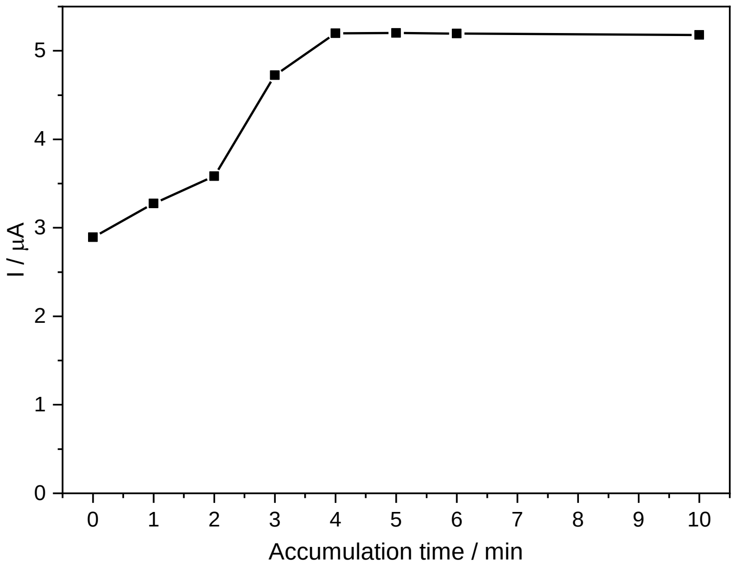

Fig. S4. Effect of accumulation time for 100 µM of PCT in PBS (0.1 M, pH 6.7).

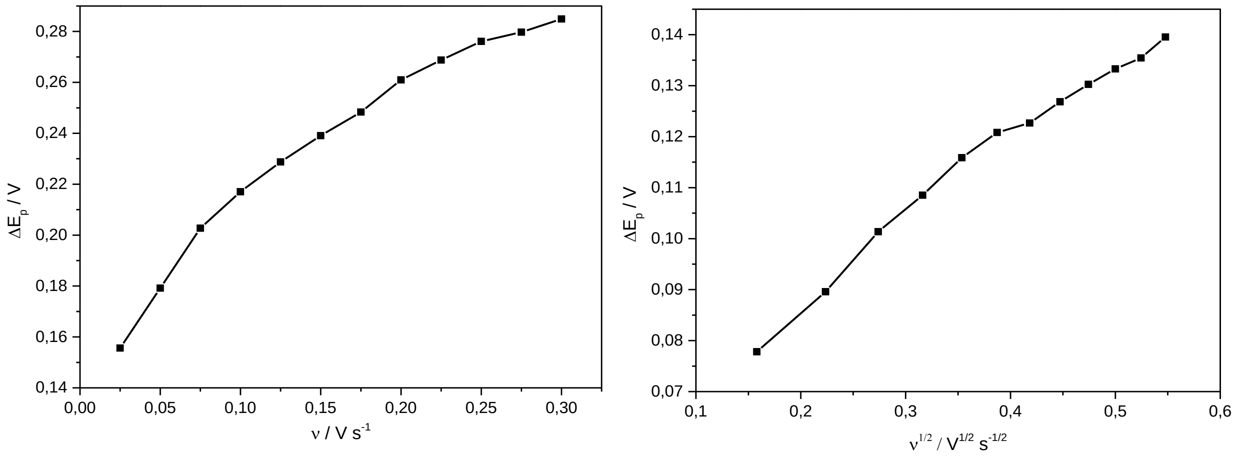

Fig. S5. Peak-to-peak separation potential vs scan rate (left) and scan rate's square root (right).

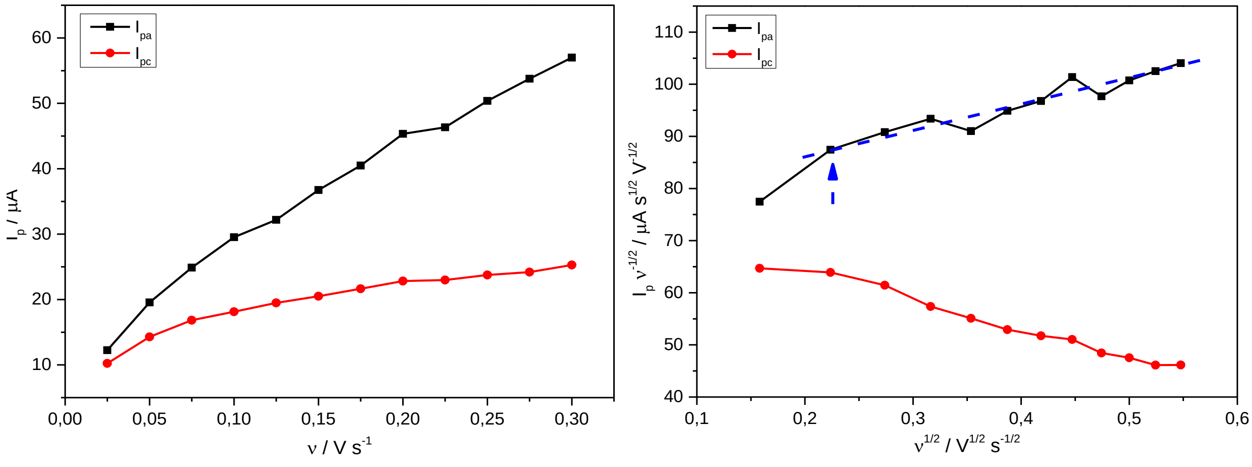

Fig. S6. Anodic and cathodic peak intensity vs scan rate (left) and anodic and cathodic peak intensity and scan rate's square root ratio ($I_p/\sqrt{v}$) vs scan rate's square root (right).

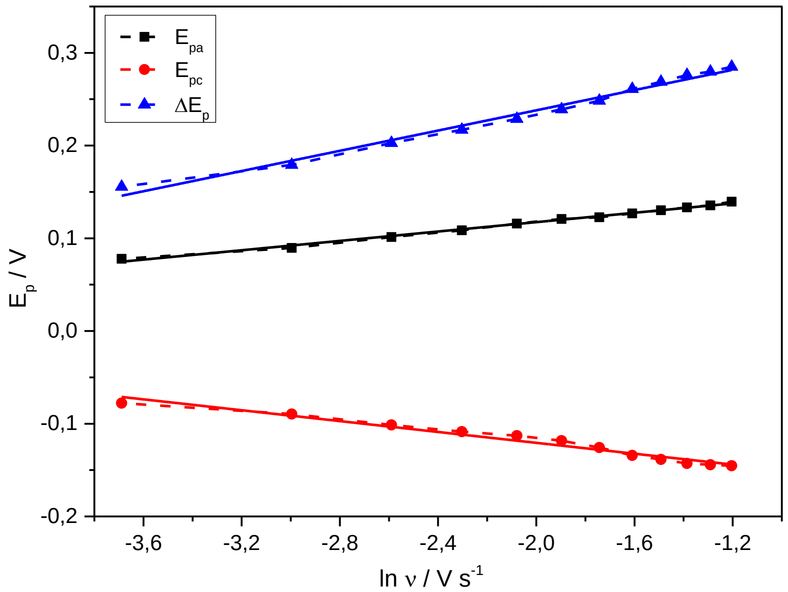

Fig. S7. Anodic and cathodic peak potentials, and peak-to-peak separation potential vs ln(*ν*).

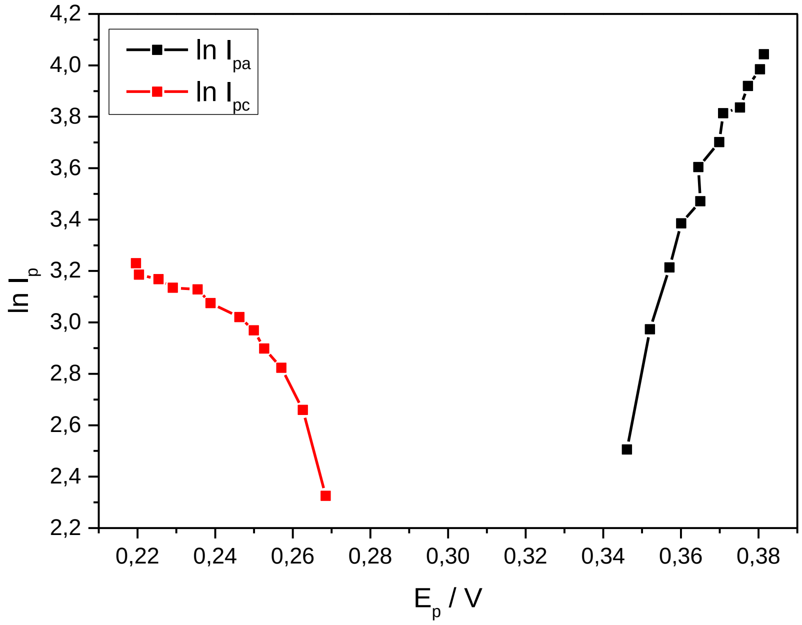

Fig. S8. Ln(I_p) vs the peak potential E_p.

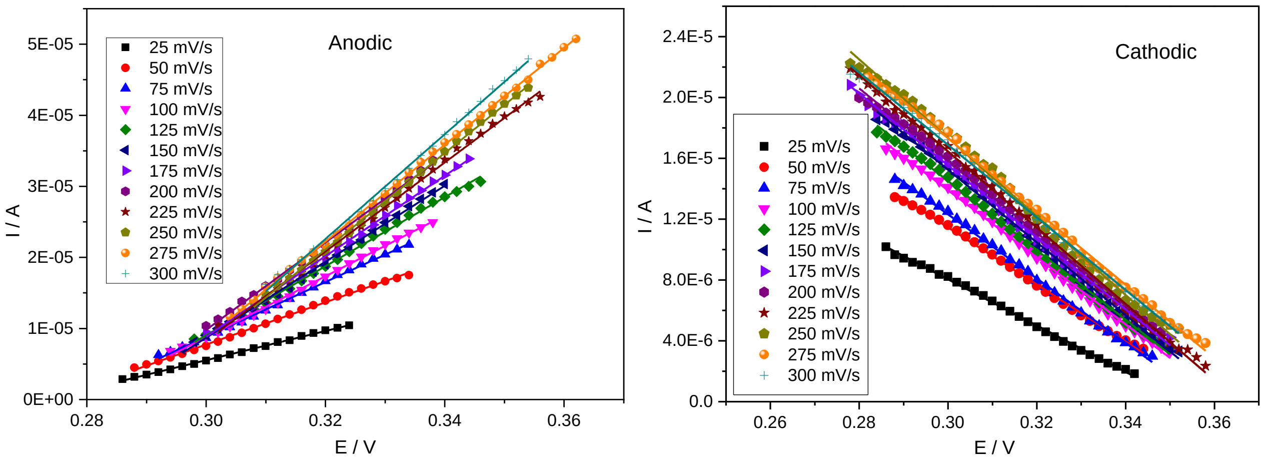

Fig. S9. Plots of intensity (anodic and cathodic) vs potential around the equilibrium situation.

Table S3. Values of *D* for the anodic (D_a) and cathodic (D_c) processes (A = 0.5 cm^2).

D_a, cm^2 s^{-1}	D_c, cm^2 s^{-1}
2.45E-05	2.41E-05
2.64E-05	2.27E-05
2.77E-05	2.20E-05
2.73E-05	2.15E-05
2.88E-05	2.19E-05
2.88E-05	2.16E-05
2.84E-05	2.13E-05
2.87E-05	2.07E-05
2.89E-05	2.18E-05
2.89E-05	2.13E-05
3.00E-05	2.14E-05
2.96E-05	2.17E-05

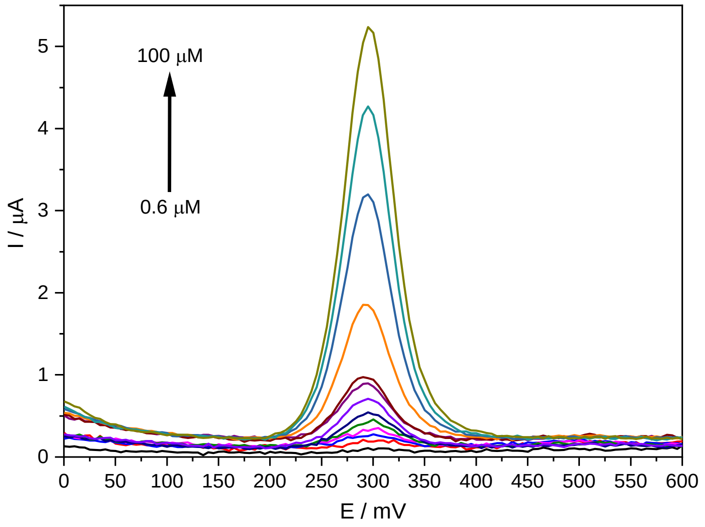

Fig. S10. Differential pulse voltammograms of various PCT concentrations [0.6 μM – 100 μM] solutions in PBS (0.1 M, pH 6.7) employing the Stv-CPE.

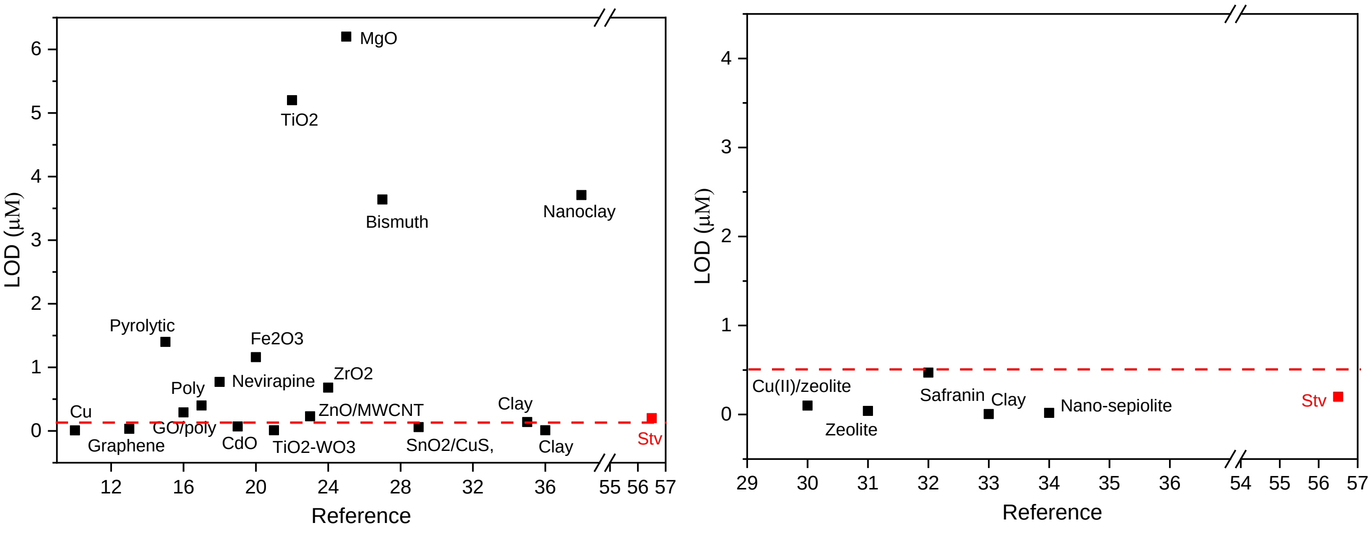

Fig. S11. LOD of several electrodes. Note the scale change in both figures.
